# Supplementary material for: Platelet-specific P2Y1 receptor deficient mice have suppressed pulmonary leukocyte recruitment in response to lipopolysaccharide
Source: Respir Res. 2026 Mar 5;27:165. doi: 10.1186/s12931-026-03611-8 (PMC13072548; doi:10.1186/s12931-026-03611-8)
Supplement: Supplementary file 1 — Supplementary Material 1. [file 12931_2026_3611_MOESM1_ESM.pdf]

Suppl Figure 1

A

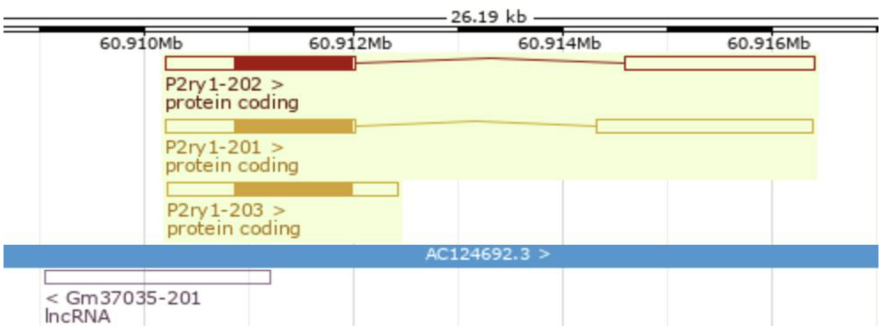

B

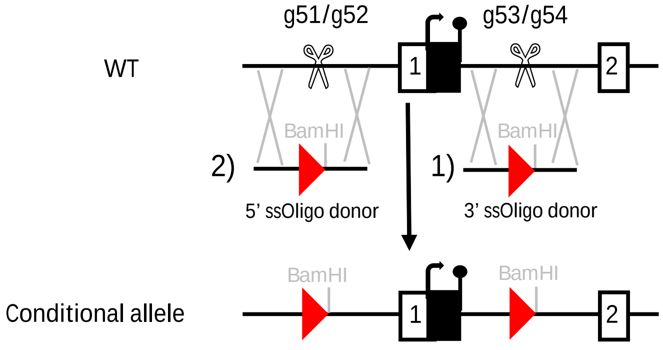

gRNA	gRNA sequence 5' --> 3'	PAM
gRNA-51	CAACTCCTTGAGTGTTTTT A	AGG
gRNA-52	AACTCCTTGAGTGTTTTTA A	GGG
gRNA-53	ATACTTCTATTCAGTAAGA A	AGG
gRNA-54	TCAGTAAGAAAGGAGATT AC	AGG

3' ssOligo donor sequence (141mer): HA-loxP-BamHI-HA

5'- GAAGCATCAATTTGATGCACATATCCACATTTATTGTTCTTTCCTGTAATCTCCTATAACTTCGTATAGCATACATTATAC
GAAGTTATGGATCCTTCTTACTGAATAGAAGTAT
GTGTAATAAACACTTCCCCTTCCTCC - 3'

5' ssOligo donor sequence (170mer): HA-loxP-BamHI-HA

5'- GGCTTGGCGGTAAGTGCCTAATGCCTTTAGTGGCTGAGACAGATCACCAACTCCTTGAGTGTTTTATAACTTCGTATAG
CATACATTATACGAAGTTATGGATCCTAAGGGAACAAA
GTCATTTAATATATGAAACTAGAATGAAATGGCTATAGAATATAATCGAAGGT 3'

C

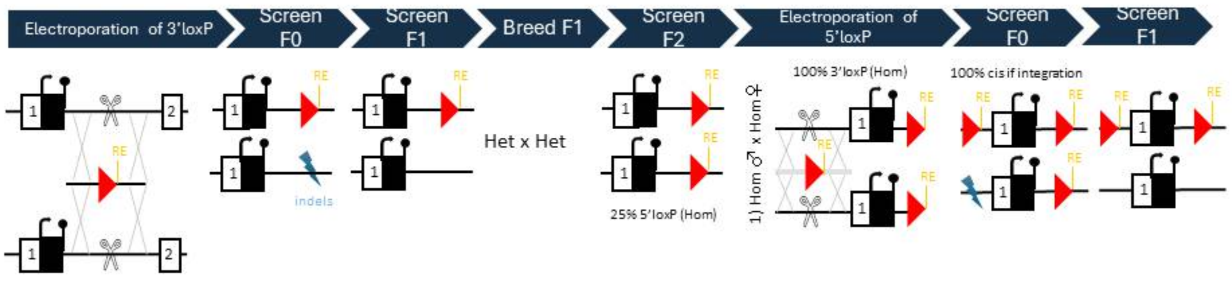

Suppl Figure 1. Creation of Platelet-P2Y1$^{-/-}$ conditional knock out mouse. Identification of transcript of interest from the Genome Reference Consortium Mouse Build 38 patch release 6 (GRCm38.p6) (**A**). Decision for location of LoxP insertion around P2Y$_1$ gene on exon 1 (**B**). Breeding strategy for electrocorporation of 3' LoxP and 5' LoxP (**C**).
