# Supplementary material for: Platelet-specific P2Y1 receptor deficient mice have suppressed pulmonary leukocyte recruitment in response to lipopolysaccharide
Source: Respir Res. 2026 Mar 5;27:165. doi: 10.1186/s12931-026-03611-8 (PMC13072548; doi:10.1186/s12931-026-03611-8)
Supplement: Supplementary file 2 — Supplementary Material 2. [file 12931_2026_3611_MOESM2_ESM.pptx]

## Slide 1
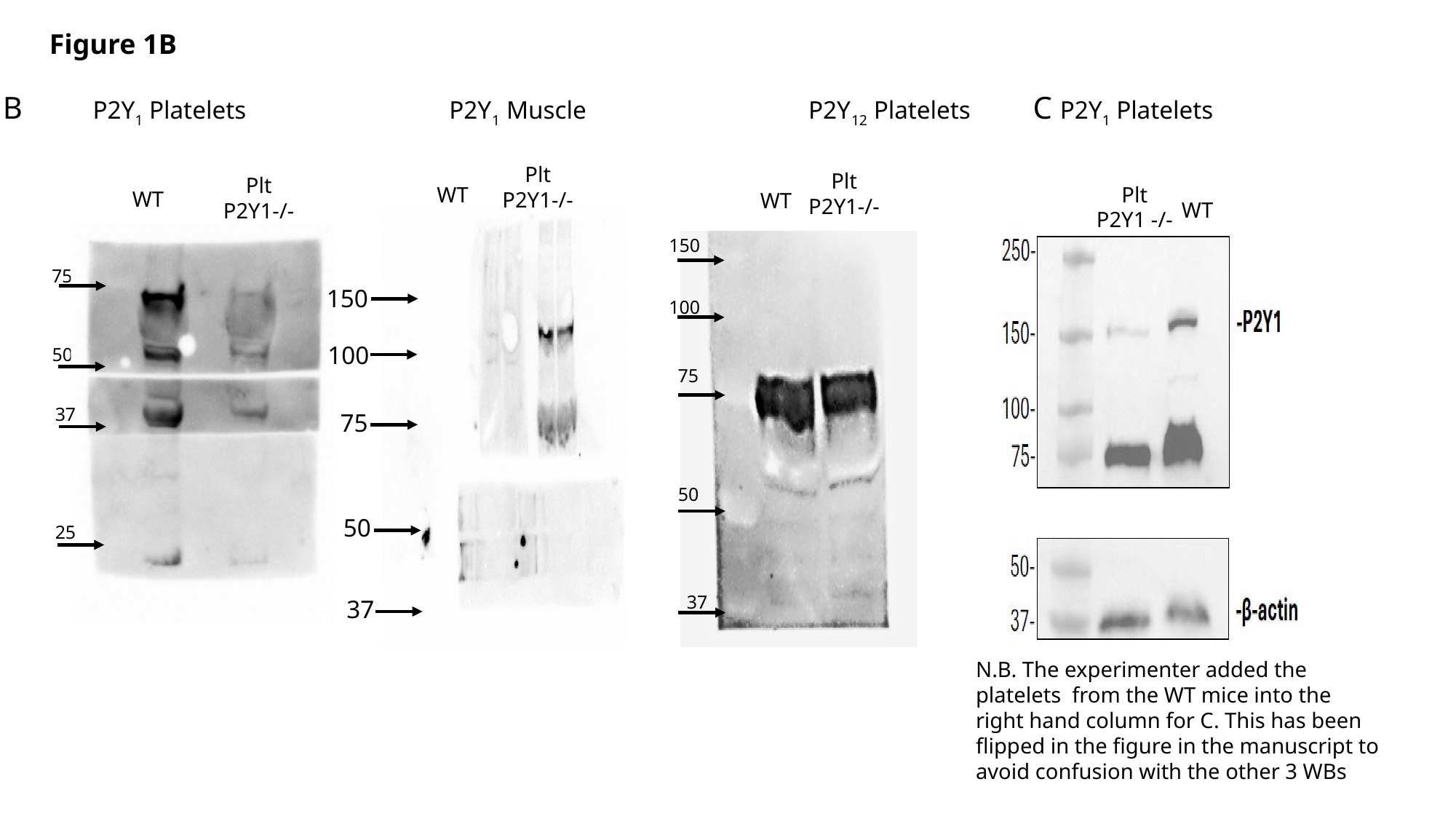

Figure 1B
B P2Y1 Platelets P2Y1 Muscle P2Y12 Platelets
C P2Y1 Platelets
Plt
P2Y1-/-
Plt
P2Y1-/-
Plt
P2Y1-/-
Plt
P2Y1 -/-
WT
WT
WT
WT
150
100
75
50
37
75
150
100
50
37
75
50
25
37
N.B. The experimenter added the
platelets from the WT mice into the
right hand column for C. This has been
flipped in the figure in the manuscript to
avoid confusion with the other 3 WBs
